# The Role of the Brain in Conscious Processes: A New Way of Looking at the Neural Correlates of Consciousness

**DOI:** 10.3389/fpsyg.2018.01346

**Published:** 2018-08-03

**Authors:** Joachim Keppler

**Affiliations:** Department of Consciousness Research, DIWISS, Roth, Germany

**Keywords:** theoretical framework for consciousness, neural correlates of consciousness, stimulus-induced conscious processes, self-referential conscious processes, memory retrieval, sense of self, stochastic electrodynamics, zero-point field

## Abstract

This article presents a new interpretation of the consciousness-related neuroscientific findings using the framework of stochastic electrodynamics (SED), a branch of physics that sheds light on the basic principles underlying quantum systems. It is propounded that SED supplemented by two well-founded hypotheses leads to a satisfying explanation of the neural correlates of consciousness. The theoretical framework thus defined is based on the notion that all conceivable shades of phenomenal awareness are woven into the frequency spectrum of a universal background field, called zero-point field (ZPF), implying that the fundamental mechanism underlying conscious systems rests upon the access to information available in the ZPF. The body of evidence can be interpreted such that in the extroverted, stimulus-oriented operating mode the brain produces streams of consciousness by periodically writing persistent information states into the ZPF (theta cycle). In the introspective operating mode, which goes along with activations of the default mode network, the brain is receptive to the flow of ZPF information states that constitutes the record of conscious experiences, suggesting that the sense of self and the retrieval of memories is accomplished by periodically reading (filtering) persistent information states from the ZPF (alpha cycle). Moreover, the data support the conclusion that meditative practices and psychedelics detune the filter, thus preventing the instantiation of self-referential conscious states, which leads to the dissolution of the ego. Instead, the brain taps into a wider spectrum of ZPF modes and, hence, gains access to an extended phenomenal color palette, resulting in expanded consciousness.

## Introduction

In the last decades, many experiments have been conducted and a huge amount of data has been collected with the goal of gaining insight into the mechanisms underlying conscious processes and paving the way for a theory of consciousness. This strategy relies on the belief of many scientists that induction is one of the core characteristics of science, implying that a theory is the end product of long sequences of experiments and observations. However, the widespread myth of induction was already dispelled by Karl Popper who discussed the processes of scientific theory formation and concluded that the “actual procedure of science is to operate with conjectures" and that “observations and experiments function in science as *tests* of our conjectures or hypotheses" (Popper, 1963).

Following this way of thinking, I begin with the formulation of hypotheses regarding conscious systems, resulting in a theoretical framework for consciousness. As we will see, this framework is ideally suited for establishing the fundamental description level at which the physical and mental aspects of our world can be merged into a coherent whole. On this basis, I present a new interpretation of the experimental findings, starting with stimulus-induced conscious processes and then, in greater detail, turning to self-referential conscious processes. In either case, we will arrive at a satisfying explanation of the neural correlates of consciousness. The article concludes with a brief discussion of the new perspectives the presented approach opens up for consciousness research. In particular, I address the future direction of experiments to test the formulated hypotheses.

## Theoretical Framework for Consciousness

In order to integrate consciousness into the scientific worldview, we resort to the most basic level of physics and follow the path of quantum theory. Only at that level will it be possible to establish a connection between consciousness and the fundamental interactions that form the foundations of physics, considering that such a connection is an indispensable prerequisite for a causally closed scientific description of our world. In this context, I would like to emphasize, however, that the selected route differs significantly from other approaches that try to establish a connection between quantum physics and consciousness. Most of these attempts (Beck and Eccles, 1992; Stapp, 1993; Hameroff and Penrose, 1996) relate consciousness to quantum state reductions in the brain and attribute the mystery of consciousness to the poorly understood transition from potentiality to actuality, without providing insight into the true nature of the mind. In contrast, the presented approach is based on stochastic electrodynamics (SED), a branch of physics that affords a look behind the scenes of quantum mechanics and quantum field theory (QFT), in particular quantum electrodynamics (QED), thus preparing the ground for a deeper understanding and explanation of quantum phenomena (Marshall, 1963, 1965; Boyer, 1969, 1975; De la Peña-Auerbach and Cetto, 1977; De la Peña and Cetto, 1994, 1995, 1996, 2001, 2006; De la Peña et al., 2009, 2015; Cetto et al., 2012, 2014). More precisely, instead of purely describing quantum systems, as the conventional formalism of QFT does, SED is capable of unveiling the mechanisms that account for the quantum behavior of matter. In this way, SED opens up new vistas that otherwise remain concealed behind the formalism of QFT.

In essence, SED is based on the conception that the universe is imbued with an all-pervasive electromagnetic background field, called zero-point field (ZPF), which, in its original form, is a homogeneous, isotropic, scale-invariant and maximally disordered ocean of energy with completely uncorrelated field modes and a unique power spectral density. According to SED, the electrically charged components of every physical system interact unavoidably with the radiative background, resulting in a stochastic oscillation of the system components. Hence, every material system can be regarded as an open stochastic system in permanent contact with the random ZPF, with each individual system responding to a specific set of relevant field modes that are selectively extracted from the full frequency spectrum of the ZPF (De la Peña et al., 2015). As long as the interaction strength between the oscillating components and these relevant field modes, for which the system exhibits a strong resonant behavior, exceeds disturbing forces such as thermal noise, the energy exchange between the system and the ZPF can reach equilibrium. Such a balance situation, in which the average power absorbed by the system compensates exactly the average radiated power, imposes restrictions on the dynamics of the system components that manifest themselves in quantization conditions in accordance with the stationary states predicted by quantum theory (De la Peña and Cetto, 1995, 2001, 2006). Expressed differently, a system in equilibrium with the ZPF falls into a dynamically stable state, i.e., an attractor, and displays quantum behavior (De la Peña and Cetto, 1995). The maintenance of the balance situation is accompanied by a modification and partial organization of the local field in such a way that the relevant ZPF modes become highly correlated (De la Peña and Cetto, 2006; De la Peña et al., 2009). In other words, the orchestration of an attractor requires the initially chaotic ZPF to change over to a partially ordered state that is characterized by an attractor-specific set of phase-locked field modes. As a result, all the components of the system are effectively coupled through the ZPF, giving rise to collective cooperation and long-range coherence (De la Peña and Cetto, 2001).

In summary, SED revolutionizes our notion of reality by giving significance to the ZPF as a creative agent that shapes matter and is the root cause of quantum phenomena. One of the key insights from SED is that quantum phenomena are emergent phenomena that can be traced back to the resonant interaction between the system components and the ubiquitous background field, which means that the properties of a quantum system are not intrinsic properties, but *dynamically acquired* properties that can be attributed to a system over the lifetime of an attractor. The phase-locked ZPF modes associated with an attractor represent a local *ZPF information state* that exhibits higher information content as compared to the disordered initial state of the background field. Each attractor features its unique ZPF information state and, hence, its specific set of phase-locked field modes *extracted* from the full frequency spectrum of the ZPF (Keppler, 2016). In combination with other works that emphasize the importance of long-term memory to explain the dynamics of quantum systems (De la Peña-Auerbach and Cetto, 1977; Fort et al., 2010; Bush, 2015), these insights support the conception of the ZPF as an *information-preserving medium* that is enriched with an ever-growing number of persistent ZPF information states.

The aforementioned characteristics and unique properties of the ZPF make one realize that this field has the potential to provide the universal basis for consciousness from which conscious systems acquire their phenomenal qualities. On this basis, I posit that *all conceivable shades of phenomenal awareness are woven into the fabric of the background field*. Accordingly, due to its disordered ground state, the ZPF can be looked upon as a formless sea of consciousness that carries an enormous range of potentially available phenomenal nuances. Proceeding from this postulate, the mechanism underlying quantum systems has all the makings of a truly fundamental mechanism behind conscious systems, leading to the assumption that *conscious systems extract their phenomenal qualities from the phenomenal color palette immanent in the ZPF*. These two hypotheses underline the dual role of the ZPF as the carrier of both energy and consciousness and express that *every ZPF information state is associated with a conscious state* (Keppler, 2012, 2013, 2016). As a consequence, conscious systems can be expected to display quantum behavior, with the accessible spectrum of conscious states of a given system being delimited by its dynamic variability, i.e., the variety of transiently stable attractors, and the quantity of consciousness of each state being determined by the degree of phase locking in the dynamically accessible part of the ZPF spectrum (Keppler, 2016). These inferences are valid unless there are plausible additional constraints for the domain of consciousness. Correspondingly, it seems reasonable to conclude that simple quantum systems, such as atoms and molecules, are equipped with a very rudimentary, limited, and monotonous form of consciousness, while complex quantum systems, such as coherent cell assemblies in the human brain, give rise to a broad range of multifaceted conscious experiences, which will be the main theme of the subsequent sections. In contrast to quantum systems, the dynamics of classical systems is completely independent of the ZPF, thus leaving the background field unaffected, preventing the generation of ZPF information states, and excluding such systems from conscious awareness.

The appeal and elegance of this approach is due to its conceptual coherence. Notably, it preserves the principle of causal closure and respects the law of parsimony, which is reflected in the idea that quantum systems acquire both their physical properties and their phenomenal qualities by use of one and the same mechanism. A pivotal feature of this mechanism is its universality, in the sense that it is available throughout the cosmos and provides access to the ubiquitous substrate of consciousness. Ensuing from this mechanism, we obtain a demarcation line between conscious and unconscious processes in such a way that *the formation of transiently stable coherent states is an essential prerequisite for conscious awareness* (Keppler, 2013, 2016, 2018).

## Stimulus-Induced Conscious Processes

As described at length in previous works, the SED-based approach provides a clear interpretation of the neuroscientific body of evidence pertaining to stimulus-induced conscious processes (Keppler, 2013, 2016, 2018). To begin with, the experimental findings indicate that the neural correlates of consciousness (NCC) are related to large-scale synchronization in the brain, particularly in the beta and gamma frequency bands (Crick and Koch, 1990; Desmedt and Tomberg, 1994; Rodriguez et al., 1999; Engel and Singer, 2001; Melloni et al., 2007; Doesburg et al., 2009; Gaillard et al., 2009; Singer, 2015). Moreover, a deeper analysis of the neurophysiological data reveals that the NCC can be equated with attractors distinguishing themselves by a high degree of coherence between spatially distributed cortical areas and that our streams of conscious perception are based on the recurring formation and dissolution of such coherent states (following the theta rhythm), with vast collections of neurons shifting simultaneously and abruptly between consecutive attractors (Freeman, 1991, 2004, 2005, 2007, 2009). These findings suggest that the NCC bear on quantum coherence since a rigorous description of the observed features, such as macroscopic pattern formation and second-order phase transitions, requires the formalism of quantum physics (Freeman and Vitiello, 2006, 2007). Most notably, the exact characteristics of critical phenomena and second order phase transitions can only be accounted for by QFT (Zinn-Justin, 1996), indicating that SED as a framework for the deeper understanding of quantum phenomena lays the foundations for explaining the dynamical properties of the NCC, particularly their rapid formation and enormous coherence length. Thus, it is postulated that the ZPF is involved in the orchestration of coherent neural activity patterns in that it is used as communication medium to establish synchronization throughout the coherence domain. Each attractor formation is accompanied by a reorganization of the ZPF, which manifests itself in the phase locking of attractor-specific field modes. According to the hypotheses formulated above, these sets of phase-locked modes are associated with conscious states, suggesting that *our brains produce our individual streams of conscious perception by periodically writing persistent information states into the substrate of consciousness* (Keppler, 2013, 2016).

**FIGURE 1:**
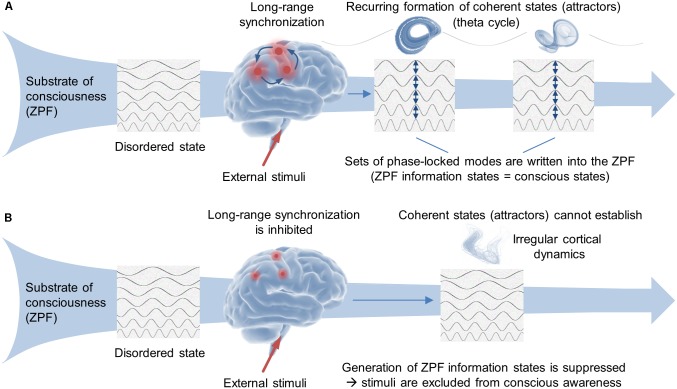
Fundamental mechanism underlying stimulus-induced conscious processes. **(A)** The neurophysiological findings strongly suggest that the ZPF is crucially involved in the orchestration of coherent neural activity patterns. Appropriate external stimuli induce second-order cortical phase transitions, resulting in the formation of attractors at theta rates. Since each attractor is associated with an attractor-specific set of phase-locked ZPF modes, this process can be interpreted in such a way that information states are periodically written into the ZPF, the presumed substrate of consciousness, thus producing our individual streams of conscious perception. **(B)** An impairment of this fundamental mechanism, occurring for instance under hypnotic conditions, inhibits the synchronization of spatially divided brain regions and, hence, the establishment of coherent long-range cortical oscillations. As a result, the formation of attractors and concomitant ZPF information states is suppressed, so that external stimuli are excluded from conscious awareness that are consciously perceived under normal conditions.

In summary, the neurophysiological findings are indicative that conscious experience depends on the integrity of this fundamental mechanism (see **Figure 1A**), while an impairment or disruption of the mechanism prevents the conscious perception of stimuli (see **Figure 1B**). Such impairment occurs for instance under hypnotic conditions where the synchronization of spatially divided brain regions is inhibited and coherent long-range cortical oscillations cannot establish (Kallio and Revonsuo, 2003; De Pascalis et al., 2004; De Pascalis, 2007; Fingelkurts et al., 2007; Miltner and Weiss, 2007; Jamieson and Burgess, 2014). As a result, the formation of attractors and concomitant ZPF information states is suppressed, so that external stimuli are excluded from conscious awareness that are consciously perceived under normal conditions (Keppler, 2018).

## Self-Referential Conscious Processes

Complementary to the extroverted, stimulus-oriented operating mode of the brain there is an introspective operating mode that goes along with activations of the default mode network (DMN), one of the resting-state networks (RSN), which has been mainly investigated using blood oxygen level dependent (BOLD) functional magnetic resonance imaging (fMRI). Several distributed cortical regions are associated with the DMN, comprising, among other areas, the precuneus, the posterior cingulate cortex (PCC), and the medial prefrontal cortex (MPFC) (Raichle et al., 2001; Greicius et al., 2003). Increased DMN activity is linked to self-referential mental processes (Gusnard et al., 2001; Buckner and Carroll, 2007), stimulus-independent thought (Mason et al., 2007), and autobiographical memory (Spreng and Grady, 2010), with the MPFC being crucially involved in memory retrieval processes (Peters et al., 2013). The dichotomy between the introspective and the stimulus-oriented operating mode is reflected in the anticorrelation between the DMN and task-positive networks, suggesting that these large-scale networks subserve opposite functions, namely stimulus-independent though on the one hand and task-focused attention on the other hand (Gusnard et al., 2001; Fox et al., 2005; Fransson, 2005; Uddin et al., 2009). Moreover, a deeper analysis of large-scale spatiotemporal BOLD signals reveals dynamical properties of the resting brain that can only be found near a critical point of a second order phase transition, indicating that, in light of the diverging correlation length, the functional connectivity between separate cortical regions is not due to propagating brain activity (Fraiman and Chialvo, 2012; Tagliazucchi et al., 2012).

The fMRI-based findings are supplemented by investigations of the electrophysiological correlates of the DMN (Knyazev, 2013), suggesting that self-referential processes are connected with enhanced alpha activity (Knyazev et al., 2011) and long-range alpha synchrony (Jann et al., 2009). Furthermore, the data point, on the one hand, to a negative correlation between DMN activity and frontal theta oscillations (Scheeringa et al., 2008) as well as a task-related suppression of gamma-band activity in the PCC and MPFC (Jerbi et al., 2010), thus corroborating the above-mentioned anticorrelation between the DMN and task-positive networks. On the other hand, they show that self-referential mental activity is associated with increased gamma power and phase synchronization in the DMN regions (Mantini et al., 2007; Knyazev, 2013). An essential insight is that the alpha rhythm becoming evident from the multichannel resting-state electroencephalogram originates from the recurring formation and dissolution of transiently stable attractors, called microstates, with each microstate remaining stable for 80–120 ms before undergoing a rapid transition to the subsequent state (Lehmann et al., 1987, 1998; Britz et al., 2010). The time series of such microstates, which represent patterns of large-scale coherent activity across the RSN areas (Khanna et al., 2015), displays scaling behavior, being an indication of self-organized criticality (Van de Ville et al., 2010).

**FIGURE 2:**
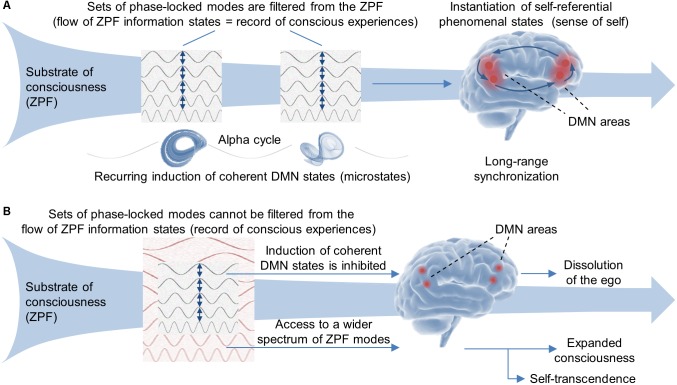
Fundamental mechanism underlying self-referential conscious processes. **(A)** The body of evidence implies that a full understanding of the dynamical characteristics of self-referential processes can only be achieved on the basis of QFT and the explanatory framework of SED. The observations can be interpreted in such a way that the sense of self is accomplished by periodically filtering persistent information states from the ZPF, the presumed substrate of consciousness. In this operating mode, the brain is receptive to the flow of ZPF information states that constitutes the record of conscious experiences, with each filtered ZPF information state inducing a cortical phase transition that results in the formation of a coherent DMN state (microstate) and the instantiation of the associated self-referential phenomenal state. **(B)** The experimental findings suggest that meditative practices and psychedelics impair this fundamental mechanism by detuning the filter. As a consequence, sets of phase-locked modes cannot be filtered from the record of conscious experiences, thus preventing the induction of coherent DMN states and the instantiation of self-referential conscious states, which leads to the dissolution of the ego. Instead, the brain gains access to a wider spectrum of ZPF modes and, hence, an extended section of the phenomenal color palette immanent in the ZPF, resulting in expanded consciousness and self-transcendence.

Taken together, these observations imply that the fundamental principles underlying self-referential processes are identical to those governing the conscious perception of external stimuli, meaning that a full understanding of their dynamical characteristics can be achieved on the basis of QFT and the explanatory framework of SED. Against this backdrop, the body of evidence, in combination with the hypotheses formulated in the second section, lends support to the conjecture that the anticorrelation between the task-positive networks and the DMN reflects the toggling between the ZPF write (recording) and ZPF read (reception) mode of the brain, suggesting that *the sense of self and the retrieval of memories is accomplished by periodically reading persistent information states from the substrate of consciousness*. More precisely, it is postulated that in the read mode the brain is receptive to the flow of ZPF information states that constitutes the record of conscious experiences, with each filtered ZPF information state inducing a cortical phase transition that results in the formation of a coherent DMN state (microstate) and the instantiation of the associated self-referential phenomenal state (see **Figure 2A**).

Notably, this interpretation is underpinned by findings regarding the impairment of self-referential processes typically found in long-term meditators who practice a “training of attention away from self-reference” (Brewer et al., 2011) and pursue the goal of “attaining a pleasant, peaceful state of mind as described in terms such as all-oneness, bliss, oceanic feeling, transcending, and expanded consciousness” (Lehmann et al., 2012). The data point to a significantly reduced activity and functional connectivity (synchrony) in the DMN regions (Brewer et al., 2011; Fingelkurts et al., 2016; Panda et al., 2016) as well as a negative correlation between the MPFC-PCC-coupling and the number of meditation hours (Marzetti et al., 2014). On the other hand, also psychedelics can induce marked subjective effects, such as “ego disintegration” (Muthukumaraswamy et al., 2013) and spiritual experiences, the common features of which include “feelings of profound joy and peace” and a “sense of oneness with the world” (Carhart-Harris et al., 2014). Experiments with psilocybin, LSD, and Ayahuasca show that the psychedelic state is characterized by substantially decreased levels of activity and functional connectivity in the DMN areas (Carhart-Harris et al., 2012, 2016; Palhano-Fontes et al., 2015). Particularly revealing is a “highly significant correlation between PCC alpha decreases and ratings of ego disturbance” together with the observation that ego dissolution goes along with broadband desynchronization and disintegration of the DMN (Muthukumaraswamy et al., 2013).

Including the fact that under the influence of psychedelics “no increases in oscillatory power were observed in any region” (Muthukumaraswamy et al., 2013), these insights suggest that the brain does not *produce* spiritual experiences. Rather, it seems eminently plausible that under normal conditions the filtering mechanism of the brain is attuned, and hence restricted, to a limited spectrum of ZPF modes, while meditative practices and psychedelics remove these restrictions by detuning the filter. As a consequence, sets of phase-locked modes cannot be retrieved from the record of conscious experiences, thus preventing the induction of coherent DMN states and the instantiation of self-referential conscious states, which leads to the dissolution of the ego. Instead, the brain *gains access to a wider spectrum of ZPF modes* and, hence, an extended section of the phenomenal color palette immanent in the ZPF, resulting in expanded consciousness and a sense of bliss and oneness with the world (see **Figure 2B**).

## Discussion

The ideas set forth in this article are based on the conceptual framework of SED, the achievement of which consists in unveiling the basic principles underlying quantum systems. It is argued that SED supplemented by two well-founded hypotheses leads to a promising theoretical approach that is able to establish a fundamental connection between physics and consciousness. The theoretical framework thus defined draws a clear demarcation line between conscious and unconscious processes, according to which the formation of transiently stable coherent states is an essential prerequisite for conscious awareness. As far as the plausibility of long-range coherence in living organisms is concerned, there are corroborating calculations on the basis of QED. These studies reveal that the special properties of water, particularly of water adjacent to hydrophilic surfaces, play an important role in the neutralization of disruptive thermal effects and that the interaction between the water molecules and the ZPF gives rise to extended coherence domains (Del Giudice et al., 2005, 2010, 2013).

As discussed in the previous sections, the SED-based approach leads to a consistent interpretation and explanation of the dynamical properties of the NCC. More precisely, the body of evidence supports the conjecture that in the extroverted, stimulus-oriented operating mode the brain produces streams of consciousness by periodically writing persistent information states into the ZPF, while in the introspective operating mode the brain accomplishes the sense of self and the retrieval of memories by periodically reading persistent information states from the ZPF. These ideas represent a *new understanding of the brain as a ZPF write-read head with a recording rate in the theta frequency band and a sampling rate in the alpha frequency band*.

Beyond its explanatory power the presented approach gives fresh impetus to the field of consciousness research in that it is able to map out a research strategy and to determine the future direction of experiments. The proposed strategy, the principal steps of which are outlined in a previous article (Keppler, 2016), amounts to a systematic calibration of ZPF information states on the basis of first-person accounts in combination with novel techniques for analyzing coherent cortical states. These techniques include photon emission spectroscopy since the recurring cortical phase transitions are expected to be accompanied by collective emissions of photons. This phenomenon, also termed superradiance, manifests itself in photon pulses (Dicke, 1954). Accordingly, in order to substantiate the proposed mechanism underlying conscious processes, the first step should be to test the prediction that such photon pulses are correlated with the theta rhythm (stimulus-induced) and the alpha rhythm (self-referential), respectively.

## Author Contributions

JK is responsible for all aspects of this article.

## Conflict of Interest Statement

The author declares that the research was conducted in the absence of any commercial or financial relationships that could be construed as a potential conflict of interest.
